# An Account of Diseases in an Eastern District of London, from April 20, to May 20, 1808

**Published:** 1808-06

**Authors:** 


*An Account of Diseases in an Eastern District of London, from April 20, to May 20, 1808.*

| | | | |
|---|---|---|---|
| ACUTE DISEASES. | | Hysteria - - - | 2 |
| Peripneumonia Notha | 4 | Hypochondriasis - | 3 |
| Ophthalmia - - | 7 | Fluor albus - - | 7 |
| Hæmoptysis - - | 3 | Menorrhagia - - | 3 |
| Rubeola - - - | 4 | Amenorrhœa - - | 4 |
| Rheumatismus Acutus | 4 | Lumbago - - | 1 |
| CHRONIC DISEASES. | | Rheumatismus Chronicus | 12 |
| Tussis - - - | 7 | PUERPERAL DISEASES. | |
| Tussis cum Dyspnœa | 10 | Ephemera - - | 5 |
| Pleurodyne - - | 3 | Mastodynia - - | 4 |
| Phthisis Pulmonalis - | 3 | Hæmorrhois - - | 3 |
| Hydrothorax - - | 2 | Dysuria - - - | 5 |
| Cephalalgia - - | 8 | INFANTILE DISEASES. | |
| Vertigo - - - | 2 | Ophthalmia - - | 5 |
| Gastrodynia - - | 2 | Ophthalmia Purulenta | 3 |
| Dyspepsia - - | 4 | Apthæ - - - | 2 |
| Icterus - - - | 1 | Tinea - - - | 2 |
| Dysuria - - - | 7 | | |

Since the very great change which has taken place in the state of the weather, the number of patients affected by complaints of the chest is considerably diminished. Those diseases, however, which were subsequent to coughs, peripneumonies, and pleurisies, and which were mentioned in the last Report, still continue to resist the most approved means of relief.

Ophthalmia has proved a troublesome, and in some instances an obstinate complaint; and though the number of patients is rather diminished, and the symptoms appear in a less aggravated form, it has not yet subsided. From the long continuance of it, an opportunity has been afforded of trying the effect of different remedies; but in this, as in in most other diseases, instead of hoping to discover any specific, the judicious practitioner finds it necesary to vary his remedies according to the symptoms which occur, and the general constitution with which they are connected. Where there has been a high degree of inflammation, general bleedings have been employed with advantage; but in lower degrees of it, the application of leeches to the temples has been found sufficient. In addition to this, a blister behind one or each of the ears, and a frequent evacuation of the bowels by a saline aperient, have been found important auxiliaries.

Saturnine lotions in some cases have been useful, and where the eye-lids have been almost closed in consequence of some morbid secretion, &c. especially when any venereal taint has been suspected, a small quantity of ung. hydrargyr. has been employed with advantage.

